# 

**Published:** 1897-03

**Authors:** 


### Mississippi Valley Dental Society.

The annual meeting of this Association will be held on Wednesday and Thursday, April 14th and 15th, in the Ohio Dental College Building, corner of Court and Central Ave., Cincinnati.

This will be the fifty-second annual meeting of this venerable society, and it is very desirable that it should be well attended and receive that regard and support from the profession which its age and past accomplishments so fully entitle it. We trust there will be a large attendance, and that all the members will come prepared to add something of interest and profit to the occasion; and those who have not been in its membership, will have an opportunity given them of being connected with this historic society. All visitors should come prepared to make this a grand meeting, and do what ever they may to promote its interest and perpetuity.

The meeting of last year was a most interesting one. Its time was devoted almost entirely to scientific professional work. This course is pursued in this body to a greater extent than in any other dental society of which we have knowledge. A very few moments of each session suffices for the transaction of routine business. Professional politics have never entered into the work of this body; it is conducted purely in the interests of the profession. Dr. J. E. Cravens, of Indianapolis, is President, and under his guidance none can question the success of the meeting. Let everybody come and bring his neighbor.

### EDITORIAL.

### The American Medical Association—Dental Section.

The semi-centennial meeting of the American Medical Association will be held in Philadelphia June 1, 2, 3, and 4, 1897. One of the important sections of this body is that of dental surgery. This section was organized about fourteen years ago, and marked an epoch in dentistry. It defined its status more definitely and better than anything that has gone before.

The section has been fairly well sustained, and much good work done in it. The work is mainly of a scientific character; clinical work has not as yet been introduced. The Committee of Arrangements announces that, in addition to the regular order of exercises during the meeting of the Association, there will be, for a week preceding and a week succeeding the Association meeting, special courses and clinics given in the various large teaching institutions of Philadelphia, without cost to visiting physicians. It is believed that many physicians from distant points would be glad to spend a week or two over and above the time occupied by the meeting in this manner. A special program for such courses will be prepared in ample time for those who desire to avail themselves of such opportunity. It is to be hoped that the dentists of Philadelphia will fall into line, in this respect, with the physicians. The dental institutions of Philadelphia would be objects of great interest to the visiting dentists. Dr. R. R. Andrews, of Cambridge, Mass., is Chairman of the section, and Dr. E. S. Talbot is Secretary.

### THE DENTAL REGISTER,

**A MONTHLY MAGAZINE DEVOTED TO THE INTERESTS OF THE**

### DENTAL PROFESSION.

*Terms Reduced to* $2.00 *Per Year. Single Copies,* 25 *Cents.*

### EDITED BY PROF. J. TAFT, D.D.S.

—AND—

### Published by SAM'L A. CROCKER & CO., Ohio Dental and Surgical Depot.

The volume commences in January and closes in December.
The REGISTER being issued monthly is always fresh, therefore Indispensable to the practitioner who desires to keep posted up with the new inventions and improvements which are daily developed. Neither expense nor effort will be spared to give value and variety to its contents. Articles will be illustrated with well executed engravings whenever they will add to the interest or assist to explanation.
Its extensive circulation and frequency of issue make it one of the BEST DENTAL ADVERTISING MEDIUMS in the United States.
All subscriptions must begin with January or July.
Local or special notices, five lines or less, will be inserted for $3 each insertion; over five lines, 50 cts. per line.
All advertising bills payable in advance, or at furthest, after the issue of the first number containing the advertisement. All transient advertisements to be paid for in advance.
☞CONTRIBUTIONS SOLICITED.
Exclusively Editorial Communications should be addressed to the Editor.
The latest number of the REGISTER may be ordered with or without other good[illegible] any time, and all letters should be addres[illegible]

